# Data on the inhibitory effect of traditional plants from Sri Lanka against tyrosinase and collagenase

**DOI:** 10.1016/j.dib.2018.08.143

**Published:** 2018-08-30

**Authors:** Jun Ito, Kotaro Hara, Takao Someya, Takao Myoda, Yoshimasa Sagane, Toshihiro Watanabe, R.G.S. Wijesekara, Kazuki Toeda, Satoshi Nojima

**Affiliations:** a Department of Food, Aroma and Cosmetic Chemistry, Faculty of Bioindustry, Tokyo University of Agriculture, 196 Yasaka, Abashiri, Hokkaido 099-2493, Japan; b ALBION Co. Ltd., 1-7-10 Ginza, Chuo-ku, Tokyo 104-0061, Japan; c Department of Aquaculture and Fisheries, Faculty of Livestock, Fisheries and Nutrition, Wayamba University of Sri Lanka, Makandura, Gonawila 60170, Sri Lanka

## Abstract

This article describes the inhibitory effects of extracts from 25 plants harvested in Sri Lanka against tyrosinase and collagenase. Inhibitors of these enzymes are common ingredients in cosmetics and medications, which help protect the skin against hyperpigmentation and premature aging. The article also discusses the polyphenol content of the extracts, which is well known to possess antioxidant properties. The extract data from the following plants, which have a long history in Sri Lankan traditional medicine, such as Ayurveda, have been provided: English name, “local name in Sri Lanka,” (scientific name). Indian copperleaf plant, “kuppameniya,” (*Acalypha indica*); red sandalwood, “madatiya”, (*Adenanthera pavonina*); balipoovu plant, “polpala,” (*Aerva lanata*); snap ginger, “heen araththa,” (*Alpinia calcarata*); bael fruit, “beli,” (*Aegle marmelos*); coastal waterhyssop, “lunuwila,” (*Bacopa monnieri*); porcupine flower, “katu karandu,” (*Barleria prionitis*); balloon-vine plant, “wel penera,” (*Cardiospermum halicacabum*); water caltrop, “Katupila,” (*Flueggea leucopyrus*); Indian sarsparilla, “iramusu,” (*Hemidesmus indicus*); malabar nut plant, “adhatoda,” (*Justicia adhatoda*); wood apple, “divul,” (*Limonia acidissima*); holy basil plant, “maduruthala,” (*Ocimum tenuiflorum*); emblic myrobalan plant, “nelli,” (*Phyllanthus emblica*); long pepper plant,”thippili,” (*Piper longum*); country borage plant, “kapparawalliya,” (*Plectranthus amboinicus*); common sesban, “wel murunga,” (*Sesbania sesban*); turkey berry, “gona batu,” (*Solanum rudepannum Dunal*); purple fruited pea eggplant,”welthibbatu,” (*Solanum trilobatum*); black plum, “madan,” (*Syzygium cumini*); crape jasmine, “wathusudda,” (*Tabernaemontana divaricate*); purple tephrosia, “pila,” (*Tephrosia purpurea*); Chinese chaste tree, “nika,” (*Vitex negundo*); and arctic snow, “suduidda,” (*Wrightia antidysenterica*). The inhibitory effects of these plant extracts on tyrosinase and collagenase, as well as polyphenol contents in the extracts, are detailed in Table 1.

**Specifications Table**

**Table:** 

Subject area	*Biology*
More specific subject area	*Food chemistry*
Type of data	*Table*
How data was acquired	*Total polyphenol content was determined by Folin-Ciocalteu method using gallic acid as a standard. Inhibitory effects of plant extracts on tyrosinase and collagenase activity were measured.*
Data format	*Analyzed*
Experimental factors	*Plant samples were harvested in the pilot farm in the Sri Lanka, and their contents were extracted with 70% ethanol.*
Experimental features	*Inhibitory effects of Sri Lankan traditional plants on tyrosinase and collagenase*
Data source location	*Negombo, Sri Lanka*
Data accessibility	*Data are available within this article*

**Value of the data**

• These data summarize the inhibitory effects of Sri Lankan plant extracts on tyrosinase and collagenase, which, because these effects are biologically important, will be valuable to a host disciplines, from the cosmetic industry, to nutrition and drug development.
• These data indicate that several plants exhibit significant inhibitory effects on tyrosinase and collagenase activities, which are important targets for future research as pharmacologic and cosmetic agents.
• These data provide a scientific assessment of plants that are widely used in Sri Lankan traditional medicine.

## 1 Data

The data, summarized in Table 1, lists the percentages of *in vitro* tyrosinase and collagenase activity in the presence of sampled plant extracts normalized against the activity of these enzymes in the absence of the extracts. The table also includes the polyphenol content of sampled plant extracts.

**Table 1:** Polyphenol content and tyrosinase and collagenase inhibitory activity for plant extracts harvested in Sri Lanka.

	Polyphenol content (mg/ml)	Tyrosinase inhibitory (%)	Collagenase inhibitory (%)
*Acalypha indica*	44.99 ± 0.46	50 ± 0.26	≦ 50
*Adenanthera pavonina*	39.91 ± 0.44	52.3 ± 0.34	≦ 50
*Adenanthera pavonina (shell)*	7.15 ± 0.07	44.23 ± 0.59	60.39 ± 5.69
*Adenanthera pavonina (fruit)*	217.24 ± 0.49	56.01 ± 1.22	≦ 50
*Aerva lanata*	38.86 ± 0.50	50.2 ± 1.44	≦ 50
*Alpinia calcarata (root)*	25.8 ± 0.48	≦ 50	97 ± 4.64
*Alpinia calcarata (leaf)*	13.80 ± 1.06	≦ 50	99 ± 3.33
*Aegle marmelos*	220.33 ± 2.57	≦ 50	≦ 50
*Bacopa monieri*	60.21 ± 0.42	≦ 50	≦ 50
*Barleria prionitis*	34.70 ± 0.80	≦ 50	≦ 50
*Cardiospermum halicacabun*	36.04 ± 0.55	≦ 50	≦ 50
*Flueggea leucopyrus*	452.27 ± 14.09	79.74 ± 0.49	77.3 ± 1.98
*Hemidesmus indicus*	86.58 ± 2.48	≦ 50	78.1 ± 4.26
*Justicia adhatoda*	96.74 ± 1.41	≦ 50	≦ 50
*Limonia acidissima*	78.72 ± 4.10	83.44 ± 1.58	≦ 50
*Ocimum ternatea*	117.44 ± 1.47	≦ 50	≦ 50
*Phyllanthus embrica* (Leaf)	173.01 ± 1.82	91.41 ± 1.27	100 ± 1.41
*Phyllanthus embrica* (Stem)	332.83 ± 1.57	100 ± 2.81	100 ± 0.31
*Piper longum*	34.356 ± 5.151	≦ 50	≦ 50
*Plectranthus amboinicus*	33.63 ± 0.19	≦ 50	≦ 50
*Sesbania sesban merr*	73.86 ± 4.53	≦ 50	≦ 50
*Solanum rudepannum* Dunal	123.00 ± 4.19	≦ 50	≦ 50
*Solanum trilobatum*	55.93 ± 9.64	≦ 50	≦ 50
*Syzygium cumini*	234.05 ± 0.45	62.3 ± 0.36	92.27 ± 2.88
*Tabernaemontana divaricate*	42.41 ± 1.15	≦ 50	≦ 50
*Tephrosia purpurea*	81.80 ± 0.56	≦ 50	≦ 50
*Vitex negundo*	298.56 ± 4.53	≦ 50	100 ± 7.52
*Wrightia antidysenterica*	52.35 ± 0.73	≦ 50	≦ 50

## 2 Experimental design, materials, and methods

### 2.1 Materials and preparation of plant extract

All plants were harvested from a medicinal garden at the Institute of Traditional Plants in Sri Lanka (Negombo, Sri Lanka). Each plant was air dried under natural conditions at about atmospheric temperature. Metabolites in the dried plants (20 g) were extracted using 150 ml of a 50% ethanol solution for 24 h. The extract was filtrated and the residues were further extracted from the filtrate for 24 h. The extracts were combined, and the solvent was removed using a rotary evaporator. The residues were then freeze-dried.

### 2.2 Determination of total phenolic content

Total phenolic content was determined by Folin-Ciocalteu method [1] using gallic acid as a standard. Sample solutions were prepared by dissolving the freeze-dried extracts in 50% methanol at concentrations of 1.0–5.0 mg/ml. The solutions (100 μl) were mixed with Folin-Ciocalteu reagent (200 μl) and then incubated for 30 min at room temperature. After adding 1 N NaOH (500 μl), absorbance at 750 nm was measured. The total phenolic content was expressed as mg gallic acid equivalents/g of sample. The assays were conducted in triplicate.

### 2.3 Tyrosinase inhibition assay

One-hundred microliters of plant extract dissolved in 50% methanol was mixed with 400 µl of 60 mM phosphate buffer (pH 6.8) and 60 µl of suspension of tyrosinase from mushroom (Sigma-Aldrich, St. Louis, MO; 30 units) and incubated at 37 °C for 20 min. Next 440 µl of 2 mM 3-hydroxy-l-tyrosine (l-DOPA) was added to the suspension and incubated at 37 °C for 5 min to allow the tyrosinase hydrolytic reaction to complete. The reaction was monitored at 475 nm. The inhibition of tyrosinase activity was calculated as: inhibition (%) = C–[S–B]/C × 100, where S is the absorbance at 475 nm in the reaction in the presence of plant extract and tyrosinase, C is the absorbance in the presence of plant extract, and B is the absorbance in the absence of tyrosinase. The assays were conducted in quadruple.

### 2.4 Collagenase inhibition assay

Twenty-five microliters of plant extract was dissolved in 50% methanol and mixed with 25 µl of suspended collagenase (Sigma-Aldrich, St. Louis, MO; 26 units/ml) in 0.1 M Tris HCl and 200 µl of 0.5 mg/ml phenylazobenzyloxycarbonyl-Phe-Leu (PZ-pepride; Bachem, Budendorf, Switzerland) solution, and incubated at 37 °C for 30 min. After the incubation, 0.5 ml of 25 mM citric acid and 2.5 ml of ethyl acetate were added to the mixture and agitated. The mixture was centrifuged (10 °C, 1500 rpm, 5 min) and the ethyl acetate layer was collected. Absorbance at 320 nm of the layer was measured, and the inhibition of collagenase activity was calculated as: inhibition (%) = C–[S–B]/C × 100, where S is the absorbance at 320 nm in the reaction in the presence of plant extract and collagenase, C is the absorbance in the presence of plant extract, and B is the absorbance in the absence of collagenase. The assays were conducted in triplicate.
