# Supplementary material for: Data on the inhibitory effect of traditional plants from Sri Lanka against tyrosinase and collagenase
Source: Data Brief. 2018 Aug 30;20:573–6. doi: 10.1016/j.dib.2018.08.143 (PMC6126212; doi:10.1016/j.dib.2018.08.143)
Supplement: Supplementary file 1 — Supplementary material [file mmc1.docx]

Conflict of Interest

All the author confirms as no Conflict of Interest.
